# Supplementary material for: Multiple myeloma long-term survivors exhibit sustained immune alterations decades after first-line therapy
Source: Nat Commun. 2024 Nov 29;15:10396. doi: 10.1038/s41467-024-54543-0 (PMC11607340; doi:10.1038/s41467-024-54543-0)
Supplement: Supplementary file 1 — Supplementary Information [file 41467_2024_54543_MOESM1_ESM.pdf]

Multiple myeloma long-term survivors exhibit sustained immune alterations decades after first-line therapy

Raphael Lutz[1,2,3,4#], Florian Grünschläger[3,4,5#], Malte Simon[5,6,7#], Mohamed H.S. Awwad[1], Marcus Bauer[8], Schayan Yousefian[9,10,11], Niklas Beumer[5,6,12,13,14], Lea Jopp-Saile[3,4,5,10,], Anastasia Sedlmeier[15], Llorenç Solé-Boldo[9,10,11], Bogdan Avanesyan[9,10,11], Dominik Vonficht[3,4,5], Patrick Stelmach[3,4], Georg Steinbuss[1], Tobias Boch[3,4,16], Simon Steiger[17], Marc-Andrea Baertsch[1,18], Nina Prokoph[1,18], Karsten Rippe[17], Brian G.M. Durie[19], Claudia Wickenhauser[8], Andreas Trumpp[3,4], Carsten Müller-Tidow[1,20], Daniel Hübschmann[3,15,21], Niels Weinhold[1,18], Marc S. Raab[1,18], Benedikt Brors[6,22,23,24*], Hartmut Goldschmidt[25*], Charles D. Imbusch[6,26,27,28*], Michael Hundemer[1*], Simon Haas[3,4,9,10,11,29*]

1. Department of Medicine V, Hematology, Oncology and Rheumatology, Heidelberg University Hospital, Heidelberg, Germany
2. Oncology Center Speyer, Speyer, Germany
3. Heidelberg Institute for Stem Cell Technology and Experimental Medicine (HI-STEM gGmbH), Heidelberg, Germany
4. Division of Stem Cells and Cancer, German Cancer Research Center (DKFZ) and DKFZ–ZMBH Alliance, Heidelberg, Germany
5. Faculty of Biosciences, Heidelberg University, Heidelberg, Germany
6. Division of Applied Bioinformatics, German Cancer Research Center (DKFZ), Heidelberg, Germany
7. Leibniz Institute for Immunotherapy (LIT), Regensburg, Germany
8. Institute of Pathology, University Hospital Halle, Martin Luther University Halle-Wittenberg, Germany
9. Berlin Institute of Health (BIH) at Charité Universitätsmedizin, Berlin, Germany
10. Berlin Institute for Medical Systems Biology, Max Delbrück Center for Molecular Medicine in the Helmholtz Association, Berlin, Germany
11. Charité Universitätsmedizin, Berlin, Germany
12. DKFZ-Hector Cancer Institute at the University Medical Center Mannheim, Mannheim, Germany
13. Department of Personalized Oncology, University Hospital Mannheim, Medical Faculty Mannheim, University of Heidelberg, Mannheim, Germany
14. Division of Personalized Medical Oncology (A420), German Cancer Research Center (DKFZ), Heidelberg, Germany
15. Computational Oncology, Molecular Precision Oncology Program, National Center for Tumor Diseases (NCT) Heidelberg and German Cancer Research Center (DKFZ), Heidelberg, Germany
16. Department of Hematology and Oncology, University Hospital Mannheim, Mannheim, Germany
17. Division of Chromatin Networks, German Cancer Research Center (DKFZ) and BioQuant, Heidelberg, Germany
18. CCU Molecular Hematology/Oncology, German Cancer Research Center (DKFZ), Heidelberg, Germany
19. Cedars-Sinai Medical Center, Los Angeles, CA, USA
20. Molecular Medicine Partnership Unit EMBL and University Hospital Heidelberg, Heidelberg, Germany

21. Innovation and Service Unit for Bioinformatics and Precision Medicine (BPM), German Cancer Research Center (DKFZ), Heidelberg, Germany
22. Medical Faculty and Faculty of Biosciences, Heidelberg University, Heidelberg, Germany
23. National Center for Tumor Diseases (NCT), Heidelberg, Germany
24. German Cancer Consortium (DKTK), Core Center Heidelberg, Heidelberg, Germany
25. Department of Medicine V, Hematology, Oncology and Rheumatology, GMMG Studygroup, Heidelberg University Hospital, Heidelberg, Germany
26. Institute of Immunology, University Medical Center Mainz , Mainz, Germany
27. Research Center for Immunotherapy, University Medical Center Mainz, Mainz, Germany
28. German Cancer Consortium (DKTK), Partner Site Frankfurt/Mainz, Mainz, Germany
29. Precision Healthcare University Research Institute, Queen Mary University of London, London, UK

# These authors contributed equally
* These authors jointly supervised this work

Correspondence should be addressed to Benedikt Brors, Hartmut Goldschmidt, Charles D. Imbusch, Michael Hundemer or Simon Haas.

E-mail: b.brors@dkfz-heidelberg.de; hartmut.goldschmidt@med.uni-heidelberg.de; c.imbusch@dkfz-heidelberg.de; michael.hundemer@med.uni-heidelberg.de; simon.haas@bih-charite.de

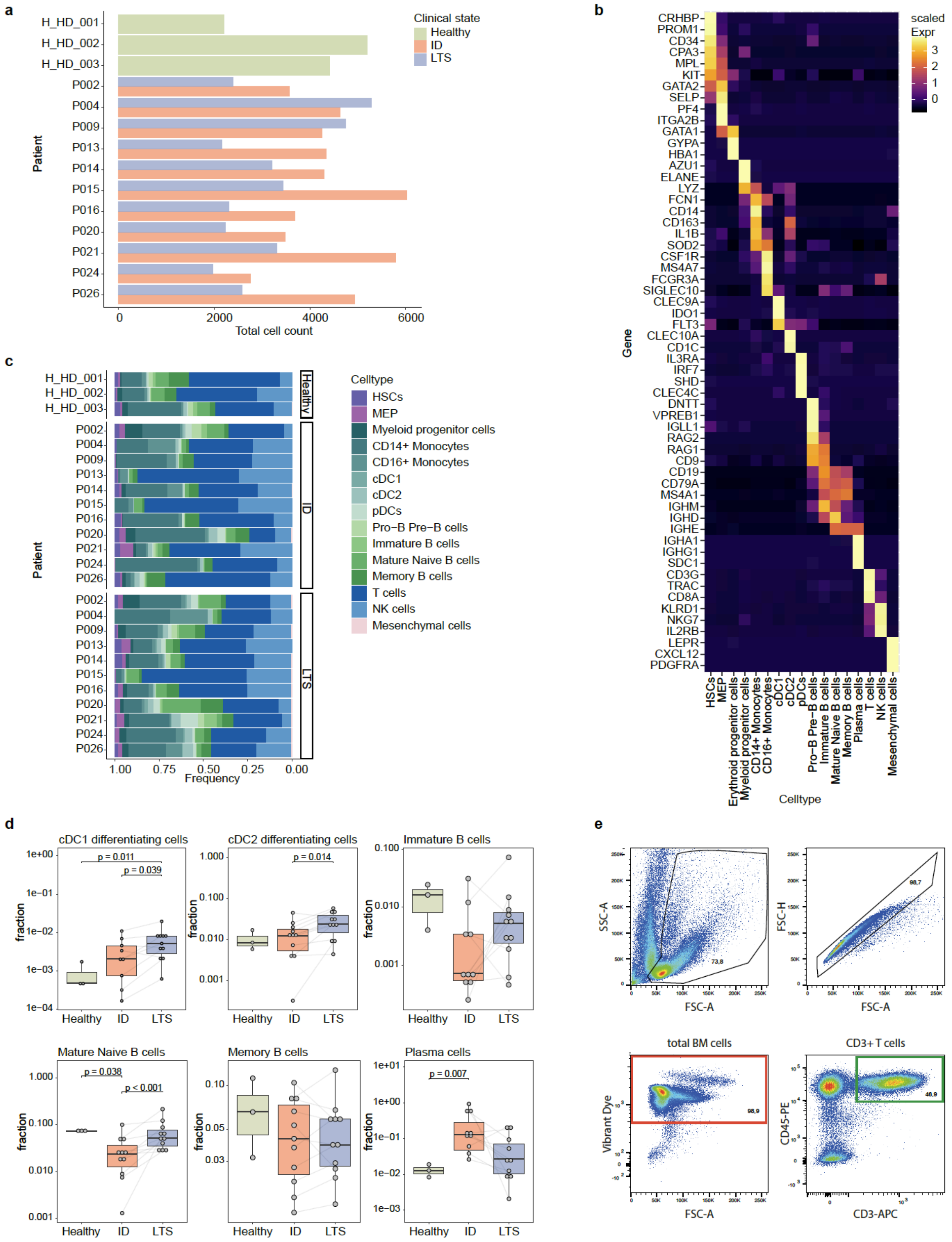

Supplementary Figure 1. Single-cell RNA-sequencing of the bone marrow immune ecosystem of multiple myeloma long-term survivor patients. (a) Total cell counts per sample and clinical state (Healthy; ID: initial diagnosis; LTS: long-term survival). **(b)** Gene expression heatmap of major marker genes for individual cell types; average gene expression per cell type, scaled row-wise for each gene. **(c)** BM cell type composition for healthy controls and MM patients per clinical condition (erythroid progenitors and plasma cells were excluded due to high variation between sample and clinical state). **(d)** Differential proportion analysis (cell type fraction of total BM cells) for conventional dendritic cells 1

and 2 (cDCs), differentiated B cell compartment (immature, mature, memory, plasma cells). Significance was tested using Wilcoxon rank sum test for unpaired comparison between healthy (n=3) and ID (n=11), or by paired Wilcoxon signed rank test between ID and LTS (n=11). **(e)** FACS gating strategy for single-cell RNA-sequencing of total BM cells and CD3+ T cells. Abbreviations: HSCs: hematopoietic stem cells, MEP: megakaryocyte-erythrocyte progenitors, MyeloP: myeloid progenitors, cDC1/2: conventional dendritic cells 1/2, pDCs: plasmacytoid dendritic cells, NK: natural killer cells, MSCs: mesenchymal stem cells; ID: initial diagnosis, LTS: long-term survival; FACS: fluorescence-activated cell sorting; SSC-A: side scatter-area; FSC-A: forward scatter-area; FSC-H: forward scatter-height. Box plots: center line, median; box limits, first and third quartile; whiskers, smallest/largest value no further than 1.5*IQR from corresponding hinge; dots: cell type fraction of total BM cells of each sample.

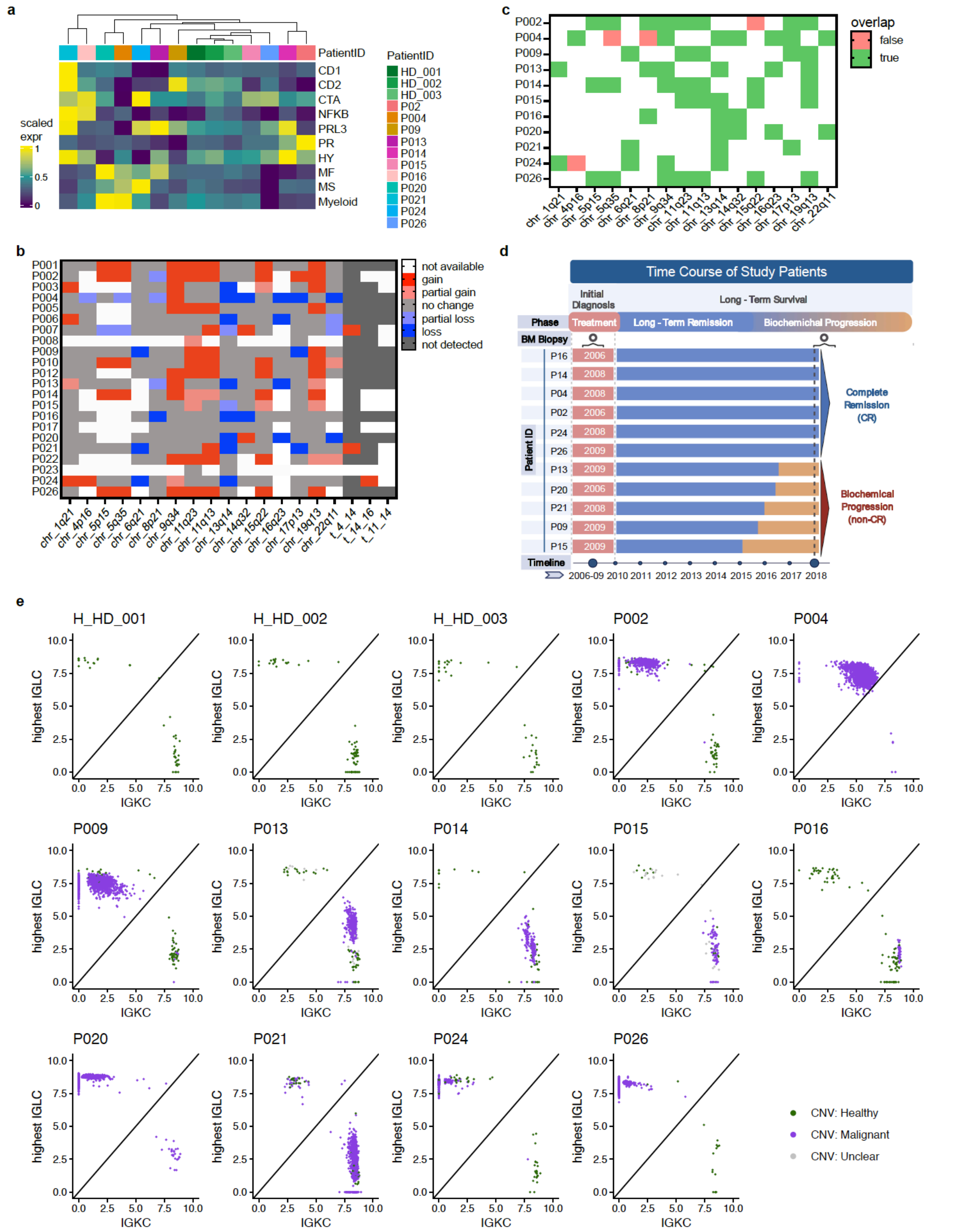

Supplementary Figure 2. Analyses of the plasma cell compartment during myeloma long-term survival. (a) Heatmap showing average expression patterns (module scores; scaled per score) of known bulk RNA-sequencing signatures per patient's plasma cells. Samples are ordered by Euclidean distance. **(b)** Copy number aberrations (CNAs) within plasma cells of each patient at ID detected by clinical routine FISH analysis. **(c)** Overlap of CNAs between standard FISH analysis and results from inferCNV (59 of 63 matches). **(d)** Time course illustration of patients subjected to scRNAseq from ID throughout LTS. **(e)** Scatterplots of immunoglobulin expression (highest lambda chain (IGLC) versus

kappa chain (IGKC)) of healthy (green) and malignant (violet) plasma cells. Abbreviations: ID: initial diagnosis; LTS: long-term survival; CNA: copy number aberrations; FISH: fluorescence in situ hybridization; IGLC: immunoglobulin light chain; LC: lambda chain; KC: kappa chain.

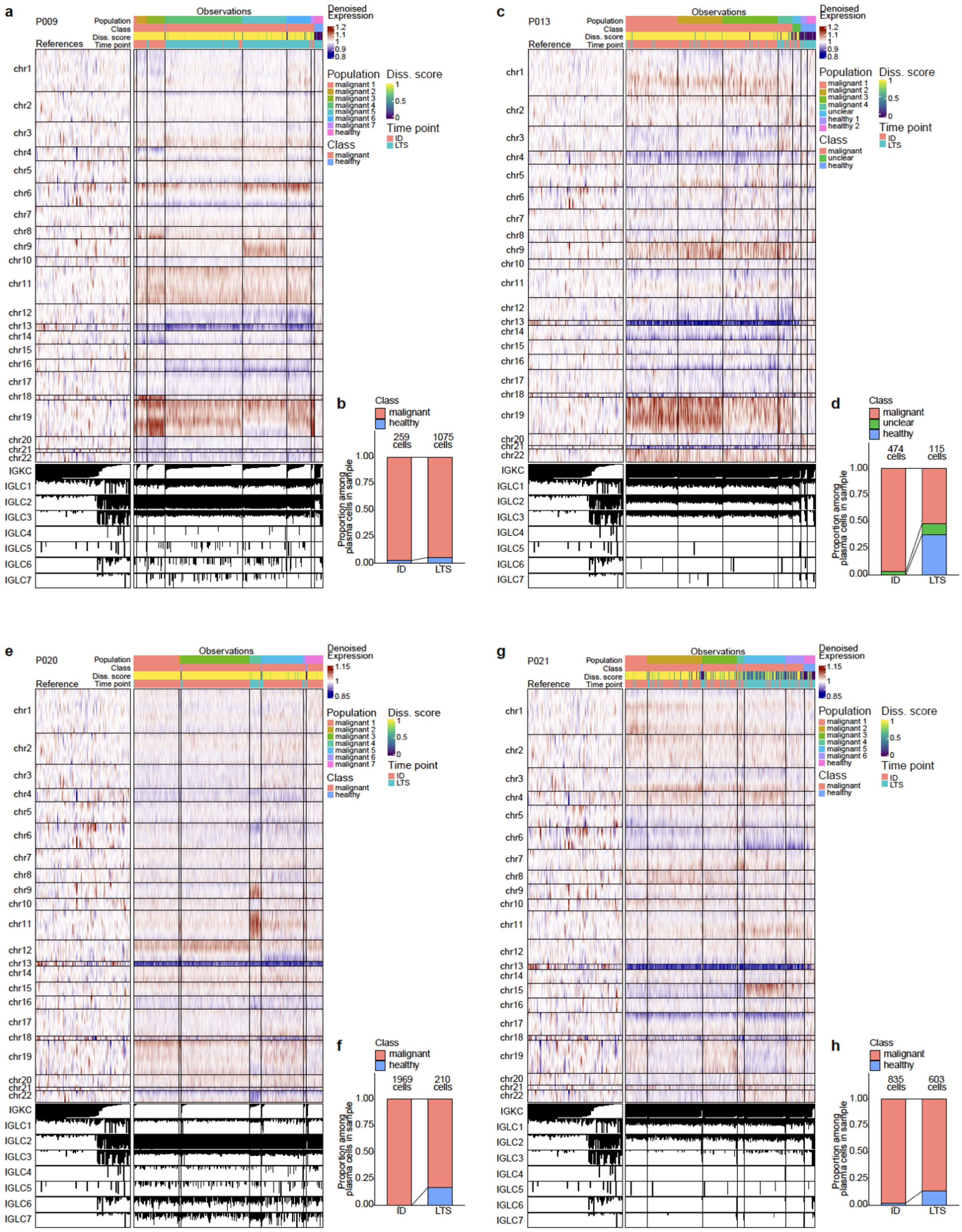

Supplementary Figure 3. Paired copy number aberration (CNA) analyses of the plasma cell compartment of MM LTS (P009, P013, P020, P021). (a,c,e,g) inverCNV-based CNA heatmaps of denoised gene expression within plasma cells of patients P009, P013, P020 and P021, respectively, compared to plasma cells from healthy controls (see methods). Only patients with sufficient numbers of PCs at both ID and LTS states are indicated. Immunoglobulin light chain expression, dissimilarity score, subclonal annotation, malignancy class and clinical state are highlighted for each cell. **(b,d,f,h)** Overall proportion of malignant (red) versus healthy (blue) plasma cells per patient (P009, P013, P020 and P021, respectively) as evaluated by inferCNV (methods).

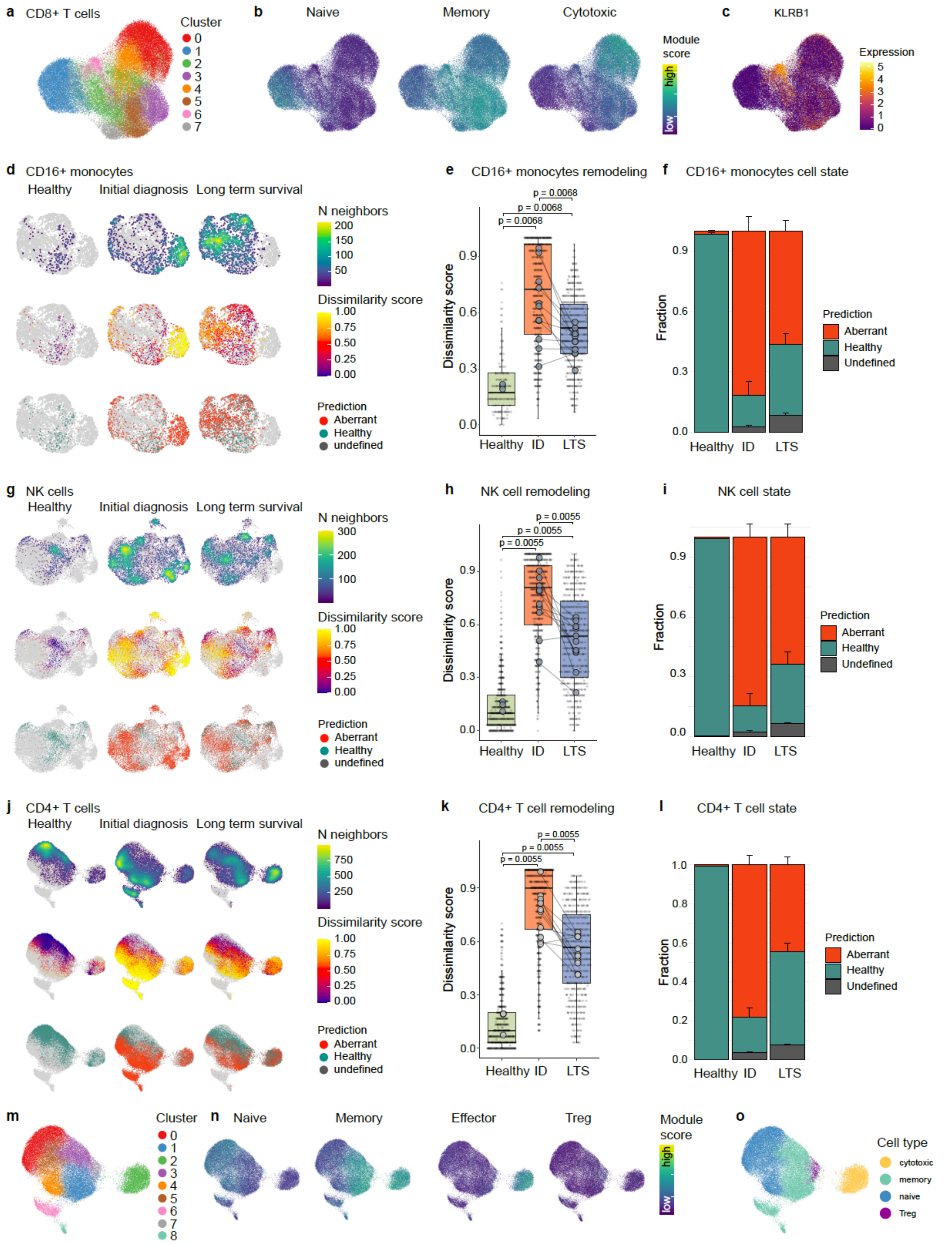

Supplementary Figure 4. Multiple myeloma long-term survivor patients display sustained signs of immune remodeling decades after a single therapy line. (a-c) CD8+ T cell dataset colored by (a) graph-based clusters, (b) module scores for naive, memory and cytotoxic CD8+ T cell gene signatures and (c) KLRB1 expression. **(d)** UMAP split by clinical groups showing cell density, dissimilarity scores and cell state predictions for CD16+ monocytes. Remaining cells from the corresponding other clinical groups are grayed out. **(e)** Boxplot of dissimilarity scores summarized by clinical groups from d. **(f)** Fractions of predicted cell states by clinical group from d. **(g-l)** Similar visualizations as in (d,e,f) are

shown for NK cells (g,h,i) and CD4+ T cells (j,k,l). **(m-o)** CD4+ T cell dataset colored by graph-based clusters (m), module scores for naive, memory and effector CD4+ T cell and Treg gene signatures (n) and CD4+ T cell subset classification based on clusters and module scores from (m,n) (o). If not stated otherwise, paired human BM samples from 11 MM patients at ID and LTS, as well as 3 healthy, age-matched controls were used for comparison. Box plots: center line, median; box limits, first and third quartile; whiskers, smallest/largest value no further than 1.5*IQR from corresponding hinge; dots: cell type fraction of total BM cells of each sample.

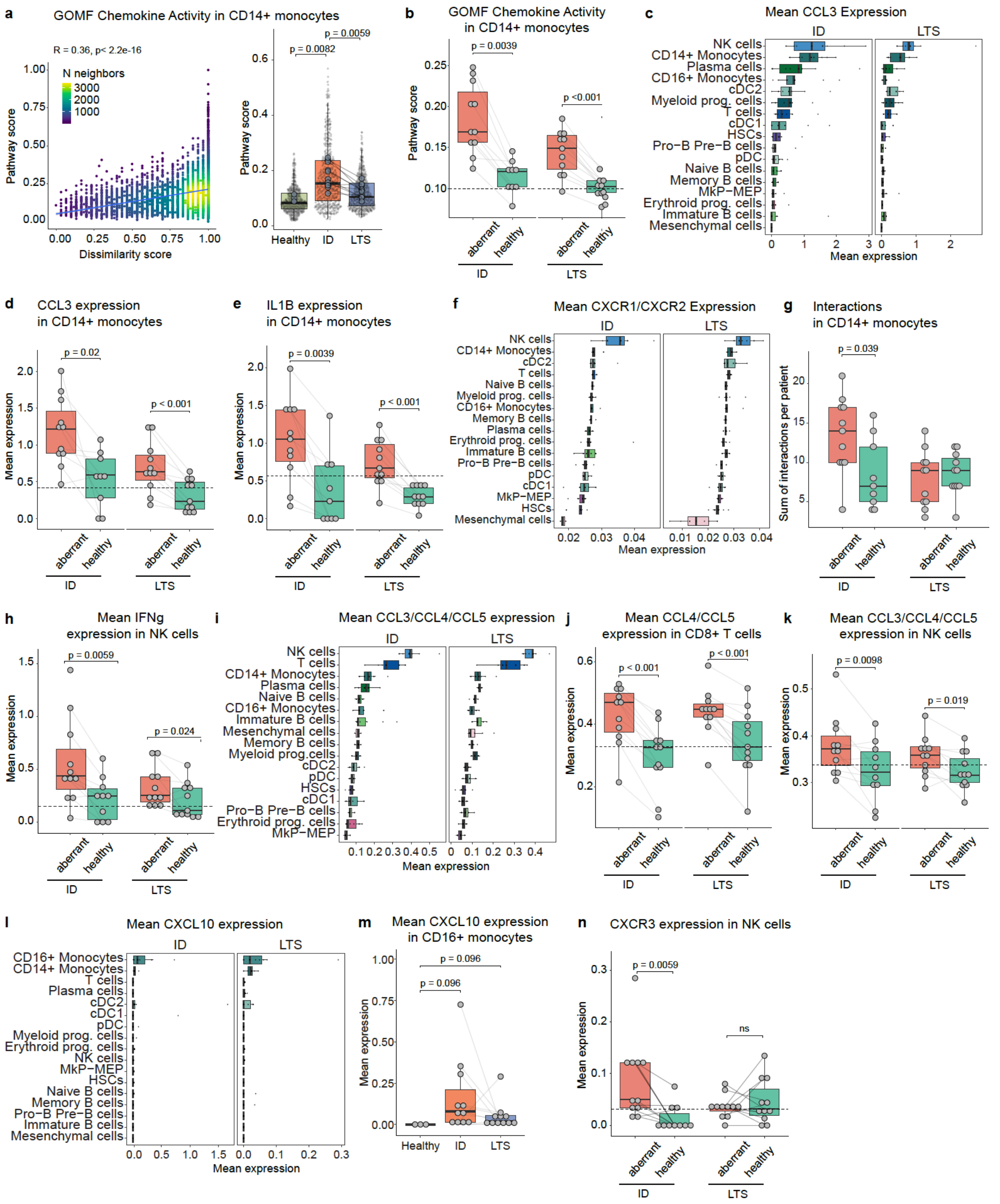

Supplementary Figure 5. An inflammatory circuit underlies immune remodeling during active disease and long-term survival. (a) Correlation of chemokine activity module score (GOMF chemokine activity) and dissimilarity score for CD14+ monocytes (left); boxplot of chemokine activity module score summarized by clinical groups (right). **(b)** Chemokine activity module score as in (a), but additionally split between predicted aberrant and healthy cells within the initial diagnosis (ID) and long-term survival (LTS) groups. **(c)** CCL3 expression summarized by cell types at ID and LTS. **(d,e)** Gene expression of CCL3 (d) and IL1B (e) in CD14+ monocytes split by clinical groups and cell state predictions. **(f)** Mean CXCR1/CXCR2 expression by cell types at ID and LTS. **(g)** Number of predicted interactions of malignant plasma cells with CD14+ monocytes (interactome analysis, see methods)

summarized by clinical group and cell state predictions. **(h)** Expression of IFNG in NK cells split by clinical groups and predicted cell states. **(i)** Mean CCL3/CCL4/CCL5 expression by cell types at ID and LTS. **(j)** Combined CCL4/CCL5 expression in CD8+ T cells grouped by clinical and cell state subsets. **(k)** Combined CCL3/CCL4/CCL5 expression in NK cells grouped by clinical and cell state subsets. **(l,m)** Mean CXCL10 expression plotted by cell types at ID and LTS (l), and for clinical groups of the CD16+ monocyte subset (m). **(n)** Expression of CXCR3 in NK cells split by clinical groups and predicted cell states. Sample sizes are as follows: n(healthy)=3, n(ID)=11, n(LTS)=11; CD14+ monocytes: n(ID/aberrant)=11, n(ID/healthy)=9, n(LTS/aberrant)=11, n(LTS/healthy)=11; NK cells: n(ID/aberrant)=11, n(ID/healthy)=10, n(LTS/aberrant)=11, n(LTS/healthy)=11; all other croups/conditions: n=11. Box plots: center line, median; box limits, first and third quartile; whiskers, smallest/largest value no further than 1.5*IQR from corresponding hinge; dots: cell type fraction of total BM cells of each sample.

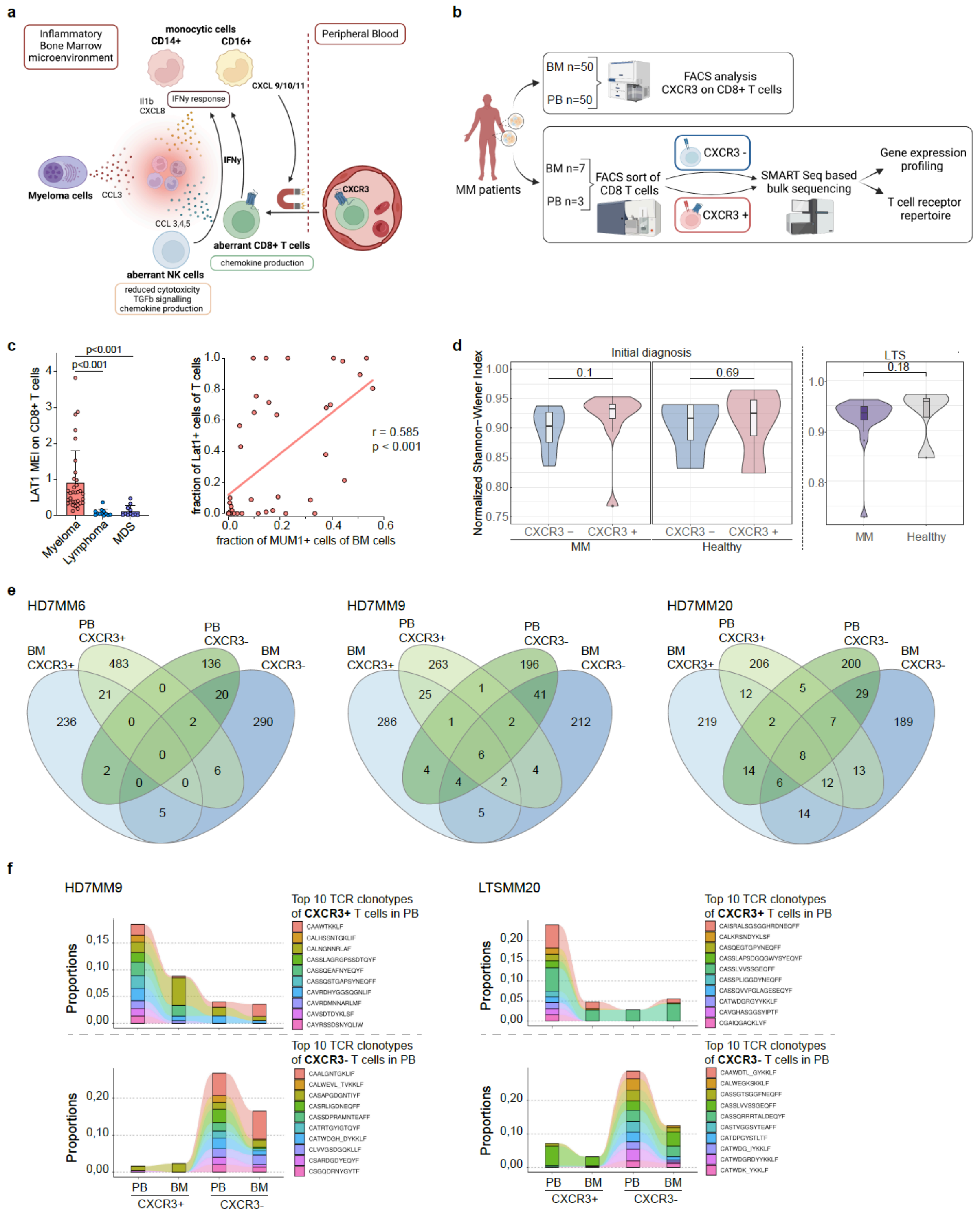

Supplementary Figure 6. Bone marrow infiltration of inflammatory T cells is associated with myeloma burden and serves as an accessible biomarker for disease activity. (a) Scheme illustrating the inflammatory circuit of aberrant immune cells in the BM microenvironment; created with BioRender.com. **(b)** Study design scheme for the comparative analysis of CXCR3-positive and -negative CD8+ T cell subsets of MM patients in PB and BM to characterize myeloma associated T cells by flow cytometry (PB: n = 50, BM: n = 50) and their TCR repertoire and transcriptome by bulk RNAseq (PB: n = 3, BM: n = 7); created with BioRender.com. **(c)** Left; LAT1 mean expression intensity (MEI) on BM CD8+ T cells during active disease state in MM, B cell non-Hodgkin lymphoma and MDS (as negative controls). Right; spearman correlation of LAT1 MEI with tumor burden measured by fraction

of MUM1+ cells in the BM. Significance was tested by unpaired Wilcoxon rank sum test and corrected using BH for multiple comparison. **(d)** Violinplot displaying the clonal diversity (normalized Shannon-Wiener Index) of TCR repertoire of CXCR3- and CXCR3+ CD8+ T cells in the BM of MM patients at ID (n = 7 patients) and healthy controls (n=4) (left), and in bulk CD8+ T cells of LTS patients (n=20) and healthy controls (n=4) (right). Significant differences were evaluated by paired Wilcoxon signed rank test. **(e)** Venn diagram highlighting overlapping TCR clonotypes by representative CDR3 amino acid sequence between CXCR3 status and sample origin (BM, PB) of CD8+ T cells of individual patients. **(f)** Clonotype tracking by representative CDR3 amino acid sequence of shared clonotypes between the top 10 most abundant TCR clonotypes from CXCR3+ (top row) and CXCR3- (bottom row) peripheral blood (PB) CD8+ T cells across CXCR3+ or CXCR3- CD8+ T cell subsets in PB and BM. Abbreviations: BM: bone marrow; PB: peripheral blood; MEI: mean expression intensity; MDS: myelodysplastic syndrome; ASCT: autologous stem cell transplantation; TCR: T cell receptor; BH: Benjamini-Hochberg.

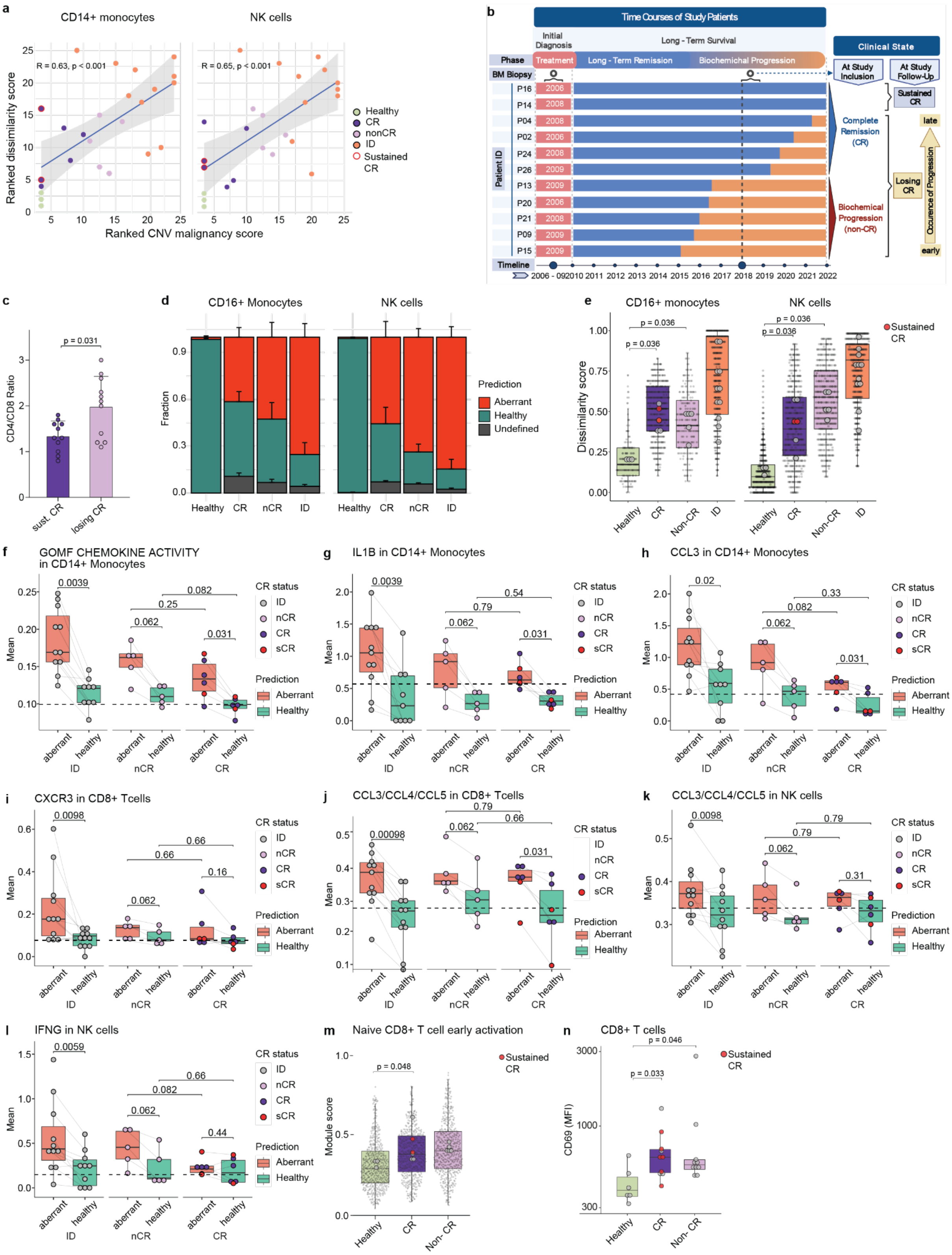

Supplementary Figure 7. Immune remodeling in LTS patients is associated with future disease resurgence and defective immune function even in the absence of measurable disease. (a) Scatterplot comparison of ranked mean dissimilarity score of CD14+ monocytes (left) and NK cells (right) versus CNV-based malignancy score (fraction of malignant plasma cells) for each patient and healthy controls; Spearman correlation was used to evaluate the relationship. **(b)** Clinical Follow up over 4 years: Time course of patients subjected to scRNAseq from ID throughout LTS including study

follow up to evaluate sustained CR. **(c)** Comparison of CD4+ to CD8+ T cell ratios between patients with sustained CR (n=11) versus patients losing CR (n=11) within peripheral blood quantified by flow cytometry; created with BioRender.com. Individual patients are highlighted as dots. Significance is shown for unpaired Wilcoxon rank sum test. **(d)** Bar plot summarizing fractions of dissimilarity-based classification into aberrant-like, healthy-like and undefined cell states by clinical group for CD16+ monocytes (left) and NK cells (right). **(e)** Distribution of the dissimilarity score (small dots) by clinical group for CD16+ monocytes (left) and NK cells (right). Large dots indicate sample means. Sustained CR patients are highlighted in red. Benjamini-Hochberg adjusted p-values from unpaired two-sided Wilcoxon rank-sum tests are shown. **(f-l)** Boxplots showing module score expression for indicated gene or gene program and indicated cell type, split by clinical group and cell state prediction. The dashed line highlights the mean module score within the healthy control group. Significant differences between aberrant and healthy cells were tested by comparing the respective sample means with paired two-sided Wilcoxon rank-sum tests. **(m)** Boxplot of module scores for the CD8 early activation gene signature from (Andreatta et al. 2021) in naive CD8+ T cells. Data is summarized and statistically tested as described in e. **(n)** Mean CD69 expression in CD8+ T cells measured by flow cytometry and compared between healthy controls (n=6), CR (n=10) and Non-CR patients (n=10) that experienced LTS. Patients in sustained CR are highlighted in red. Significance was tested using two-sided, unpaired Wilcoxon rank sum test and corrected according to Benjamini-Hochberg. If not stated otherwise, paired human BM samples from 11 MM patients at ID and LTS, further divided into CR (n=6) and nCR (n=5), as well as 3 healthy, age-matched controls were used for comparison. Abbreviations: ID: initial diagnosis; LTS: long-term survival; CR: complete remission; NK: natural killer; BH: Benjamini Hochberg. Box plots: center line, median; box limits, first and third quartile; whiskers, smallest/largest value no further than 1.5*IQR from corresponding hinge; dots: cell type fraction of total BM cells of each sample.

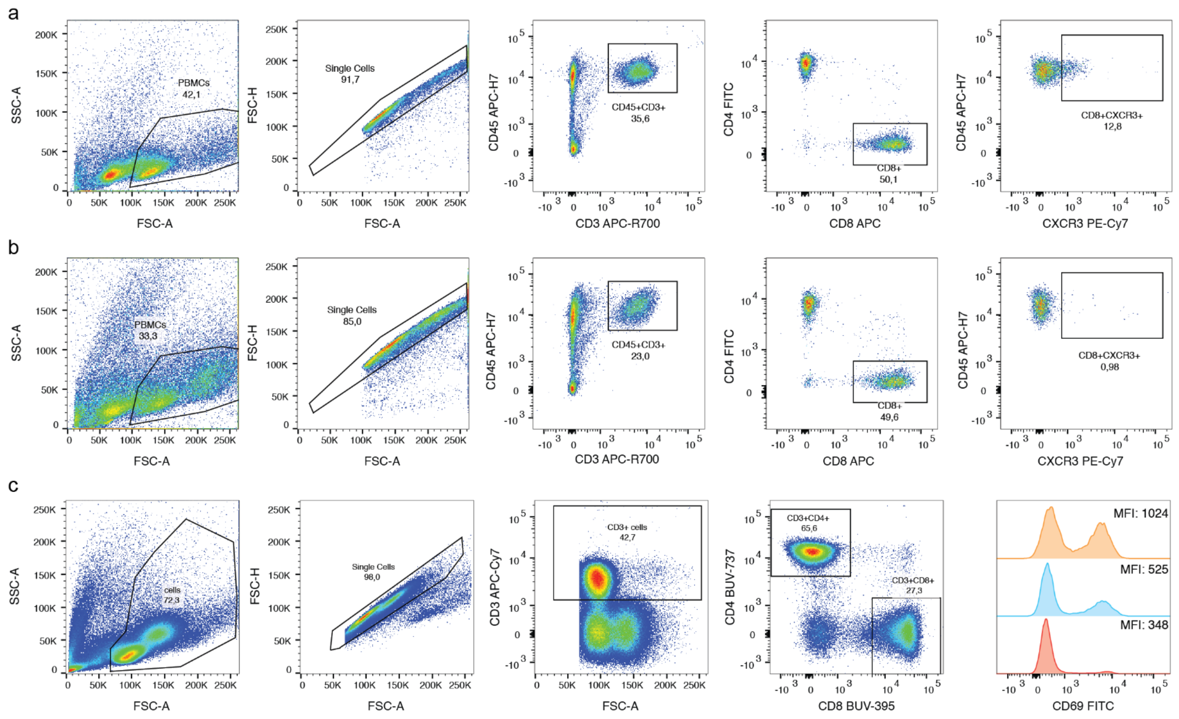

Supplementary Figure 8. Gating strategies for CXCR3 and CD69 expression. (a,b) Gating strategies from a representative patient sample harboring >10% CXCR3+ CD8+ T cells (a) and from a representative patient sample with low amount of CXCR3+ CD8+ T cells (b). **(c)** Representative gating strategy for CD69 MFI analysis of CD8+ T cells. Histogram highlights examples of low (red), intermediate (blue) and high (yellow) CD69 MFI.

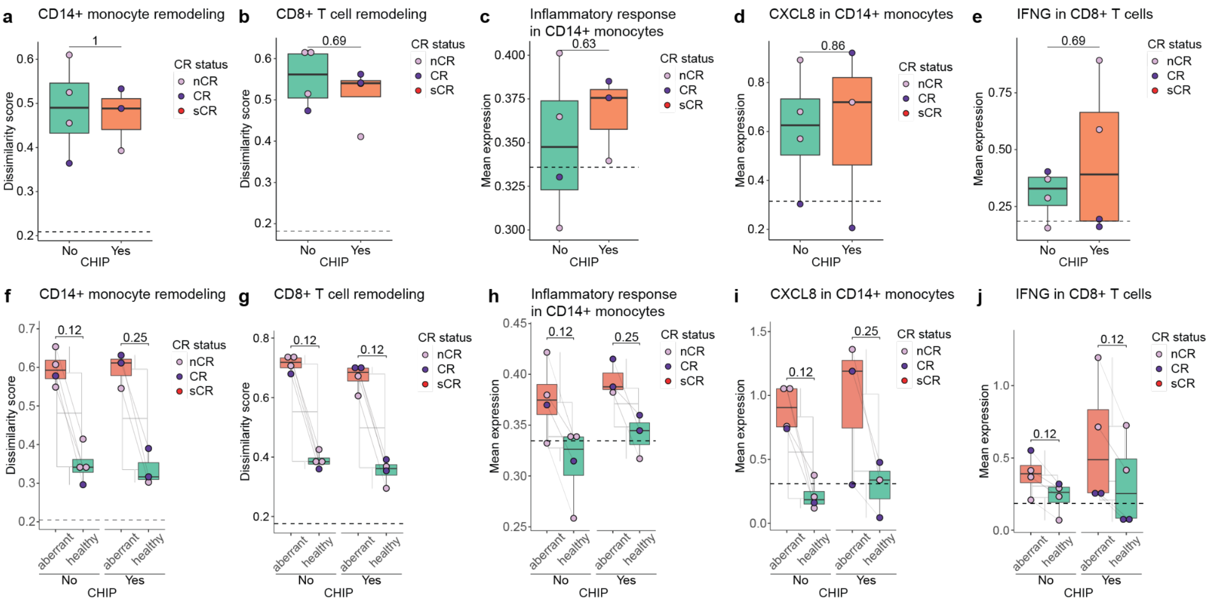

Supplementary Figure 9. Clonal hematopoiesis (CHIP) analysis. (a-e) Boxplots illustrating differences in immune remodeling (dissimilarity score) and expression patterns in CD14+ monocytes and CD8+ T cells between patients with or without detected CHIP at LTS. **(f-j)** Boxplots highlighting differences in immune remodeling and expression of genes or modules within patients split by CHIP status and cell state prediction at LTS. The dashed line highlights the mean module score within the healthy control group. Significant differences between aberrant and healthy cells were tested by comparing the respective sample means with paired two-sided Wilcoxon rank-sum tests. CR: complete remission, nCR: non-complete remission, sCR: sustained complete remission.
