# Supplementary material for: Multiple myeloma long-term survivors exhibit sustained immune alterations decades after first-line therapy
Source: Nat Commun. 2024 Nov 29;15:10396. doi: 10.1038/s41467-024-54543-0 (PMC11607340; doi:10.1038/s41467-024-54543-0)
Supplement: Supplementary file 3 — Description of Additional Supplementary Files [file 41467_2024_54543_MOESM3_ESM.pdf]

Description of Additional Supplementary Files

Supplementary Data 1.
Metadata of MM patients.

Supplementary Data 2.
Metadata of healthy individuals.

Supplementary Data 3.
Input parameters for model. Input parameters for chosen model and prediction statistics for aberrant cell type classification using H2O (see methods).
