# Supplementary material for: Multiple myeloma long-term survivors exhibit sustained immune alterations decades after first-line therapy
Source: Nat Commun. 2024 Nov 29;15:10396. doi: 10.1038/s41467-024-54543-0 (PMC11607340; doi:10.1038/s41467-024-54543-0)
Supplement: Supplementary file 7 — Reporting Summary [file 41467_2024_54543_MOESM7_ESM.pdf]

nature portfolio

Corresponding author(s): Benedikt Brors, Hartmut Goldschmidt, Charles D. Imbusch, Michael Hundemer, Simon Haas

Last updated by author(s): May 8, 2024

Reporting Summary

Nature Portfolio wishes to improve the reproducibility of the work that we publish. This form provides structure for consistency and transparency in reporting. For further information on Nature Portfolio policies, see our Editorial Policies and the Editorial Policy Checklist.

Statistics

For all statistical analyses, confirm that the following items are present in the figure legend, table legend, main text, or Methods section.

n/a	Confirmed	
☐	☒	The exact sample size (*n*) for each experimental group/condition, given as a discrete number and unit of measurement
☒	☐	A statement on whether measurements were taken from distinct samples or whether the same sample was measured repeatedly
☐	☒	The statistical test(s) used AND whether they are one- or two-sided<br>*Only common tests should be described solely by name; describe more complex techniques in the Methods section.*
☐	☒	A description of all covariates tested
☐	☒	A description of any assumptions or corrections, such as tests of normality and adjustment for multiple comparisons
☐	☒	A full description of the statistical parameters including central tendency (e.g. means) or other basic estimates (e.g. regression coefficient) AND variation (e.g. standard deviation) or associated estimates of uncertainty (e.g. confidence intervals)
☐	☒	For null hypothesis testing, the test statistic (e.g. *F*, *t*, *r*) with confidence intervals, effect sizes, degrees of freedom and *P* value noted<br>*Give P values as exact values whenever suitable.*
☒	☐	For Bayesian analysis, information on the choice of priors and Markov chain Monte Carlo settings
☐	☒	For hierarchical and complex designs, identification of the appropriate level for tests and full reporting of outcomes
☐	☒	Estimates of effect sizes (e.g. Cohen's *d*, Pearson's *r*), indicating how they were calculated

Our web collection on statistics for biologists contains articles on many of the points above.

Software and code

Policy information about availability of computer code

Data collection	Raw single-cell RNA sequencing data of BMMCs form healthy controls and MM patients was generated and processed using 10x Genomics single-cell RNAseq technology (Chromium Single Cell 3' Solution v2; Cellranger Count v4.0) according to the manufacturer's recommendations. Detailed description of data collection and processing can be found in the method section "Single-cell RNA sequencing and data preprocessing".<br>Bulk RNA sequencing libraries were generated using the SMART Seq Stranded Total RNA-Seq kit (Takara) according to manufacturer guidelines and processed using Skewer (v.0.2.2) for adapter trimming and STAR (v2.5.2b) for alignment. A detailed description can be found in the method section "Bulk RNA-sequencing and gene expression analysis".<br>Data collection for spatial information on T and plasma cells was performed by multispectral imaging using the PerkinElmer Vectra Polaris platform. Please see method section "Multiplex Immunofluorescence" for more details.<br>For the acquisition of flow cytometry data, BD FACSFortessa flow cytometer, or BD FACSLyric flow cytometer was used with BD FACSDiva software.

Data analysis	RNA sequencing data was analyzed using R v4.0.0, Python v3.9.7 and following packages and pipelines: Seurat v4.0.1, celda v1.4.0, infercnv v1.6.0, JAGS v4.3.0, SingleR v1.2.4, DA-seq, FNN v1.1.3, H2O v3.36.0.3, caret v6.0-91, Scanorama, MAST v1.19.0, fgsea v1.14.0, clusterProfiler v3.16.1, CellphoneDB2.0, scVelo c0.2.4, Velocyto v0.17.17, DESeq2 v1.30.1, MixCR v3.0.13, VDJtoos v1.2.1, immunarch v0.6.6. Details can be found in the methods section.<br>Cell segmentation and phenotyping of the cell subpopulations were performed using the inForm software (PerkinElmer Inc., USA).<br>Flow cytometry data was analyzed using FACSDiva and FlowJo v10.7.1 (BD Biosciences, San Jose, CA, USA), Infinicyt 2.0 (Cytognos, Salamanca, Spain)<br>Graphical representation and statistics where either performed using R, Python, or GraphPad Prism v9.3.1.

For manuscripts utilizing custom algorithms or software that are central to the research but not yet described in published literature, software must be made available to editors and reviewers. We strongly encourage code deposition in a community repository (e.g. GitHub). See the Nature Portfolio guidelines for submitting code & software for further information.

Data

Policy information about availability of data

All manuscripts must include a data availability statement. This statement should provide the following information, where applicable:

- Accession codes, unique identifiers, or web links for publicly available datasets
- A description of any restrictions on data availability
- For clinical datasets or third party data, please ensure that the statement adheres to our policy

The scRNAseq and bulk RNAseq data generated in this study have been deposited in the European Genome-Phenome Archive (EGA) under accession code EGAS00001006980 https://ega-archive.org/search/EGAS00001006980. The processed scRNAseq data are available at Figshare https://doi.org/10.6084/m9.figshare.26935744.

Research involving human participants, their data, or biological material

Policy information about studies with human participants or human data. See also policy information about sex, gender (identity/presentation), and sexual orientation and race, ethnicity and racism.

Reporting on sex and gender	For scRNAseq and FACS patient data, please see Table 1 & 2: "Characteristics of patients with Multiple Myeloma in LTR".<br>For bulkRNAseq patient data:<br>Bone Marrow and Peripheral Blood:<br>HD7MM6 female 65-69 years<br>HD7MM9 male, 65-69 years<br>LTSMM20, female 70-74 years<br>Bone Marrow only:<br>MM006 male, 55-59 years<br>MM007 female, 45-49 years<br>MM013 female, 40-44 years<br>MM017 male, 65-69 years<br>P656_P08 male, 50-54 years<br>P692_P23 female, 70-74 years<br>H013 male 40-44 years<br>H016 female 20-24 years<br>Hreg female 65-69 years<br>lymph14 male 60-64 years
Reporting on race, ethnicity, or other socially relevant groupings	NA
Population characteristics	Detailed information on patient characteristics of MM LTS patients (gender, age, MM type, CRAB criteria, ISS stage, cytogenetics, % plasma cell infiltration), induction treatment, conditioning, maintenance therapy and duration, pre-ASCT response, post-ASCT response, relapse after 2018 and sustained complete remission until 2022) can be derived from Table 1: "Characteristics of patients with Multiple Myeloma in LTR".<br><br>bulkRNAseq patient data:<br>Healthy controls:<br>H013,<br>H016,<br>Hreg,<br>lymph14<br><br>Multiple myeloma patients:<br>HD7MM6<br>HD7MM9<br>LTSMM20<br>MM006<br>MM007<br>MM013<br>MM017

P656_P08
P692_P23

Recruitment	Healthy donors were recruited from age-matched healthy volunteers without any clinical signs of disease.<br>Myeloma patients were recruited upon initial diagnosis.<br>Myeloma LTS patients were selected based on their long-term survival status from 7 to 17 years (median 10.5 years) after first line therapy with standard induction regimen and high dose therapy followed by autologous stem cell transplantation.
Ethics oversight	Human samples from healthy and diseased donors were obtained at Heidelberg University hospital after informed written consent using ethic application numbers S-480/2011 and S-052/2022.<br>All experiments involving human samples were approved by the ethics committee of the Heidelberg University hospital and were in accordance with the Declaration of Helsinki.

Note that full information on the approval of the study protocol must also be provided in the manuscript.

Field-specific reporting

Please select the one below that is the best fit for your research. If you are not sure, read the appropriate sections before making your selection.

☒ Life sciences ☐ Behavioural & social sciences ☐ Ecological, evolutionary & environmental sciences

For a reference copy of the document with all sections, see nature.com/documents/nr-reporting-summary-flat.pdf

Life sciences study design

All studies must disclose on these points even when the disclosure is negative.

Sample size	The number of recruited patients was not pre-determined statistically, but relied on availability of patients matching the recruitment criteria.<br>No statistical method was used to determine sample sizes for scRNAseq. Instead, prior experience and upper-bound limitations regarding feasible sample volumes and cell loading thresholds suggested an isolation of around 10,000 cells per patient.
Data exclusions	scRNAseq BM data: Cells were retained if they had between 200 – 40,000 UMIs, between 400 – 6,000 features and less than 10% mitochondrial reads. Clusters with contaminating gene expression profiles, or aberrantly high mitochondrial and low housekeeping gene expression were considered as doublets, or low quality and were removed. Cells from a patient P002 treated with maintenance and induction therapy were removed, as the sample size (n=1) was too small to draw biological conclusions.<br>Plasma cells from patient P015 were excluded for cellular abundance analysis, as only the negative MACS fraction for plasma cell enrichment was available for single cell RNA sequencing.<br>scRNAseq of T cells: Cells were retained in the dataset if they had between 500 - 20000 UMIs, between 300 - 4000 detected features and less than 10% mitochondrial reads. Clusters of contaminating cells including myeloid cells, erythroid progenitors, plasmablasts and cycling cells were excluded prior to downstream analyses.<br>bulkRNAseq samples P656_P08 and P692_P23 were excluded for clonotype analysis due to insufficient number of aligned reads for TCR clonotype assembly.
Replication	The number of biological replicates used in each experiment is indicated throughout the manuscript. For single cell RNA seq, 11 independent multiple myeloma LTS patients were assayed.
Randomization	Not applicable
Blinding	No blinding was used

Reporting for specific materials, systems and methods

We require information from authors about some types of materials, experimental systems and methods used in many studies. Here, indicate whether each material, system or method listed is relevant to your study. If you are not sure if a list item applies to your research, read the appropriate section before selecting a response.

Materials & experimental systems

n/a	Involved in the study
☐	☒ Antibodies
☒	☐ Eukaryotic cell lines
☒	☐ Palaeontology and archaeology
☒	☐ Animals and other organisms
☒	☐ Clinical data
☒	☐ Dual use research of concern
☒	☐ Plants

Methods

n/a	Involved in the study
☒	☐ ChIP-seq
☐	☒ Flow cytometry
☒	☐ MRI-based neuroimaging

Antibodies

Antibodies used

FACS antibodies (Epitope+Fluorochrome, Vendor + Catalog number, RRID, clone, dilution):

Single cell Sorting panel:
Anti-CD45-PE, Biolegend Cat# 304058, RRID: AB_2564156 ,HI30, 1:50
Anti-CD3-APC, BioLegend Cat# 300312, RRID: AB_314048 , HIT3a, 1:10
Vibrant DyeCyle Violet stain, Thermo Fisher Scientific Cat# V35003, (see manufacturer's instructions)

MRD analysis
Multiple Myeloma MRD Kit, Cytognos, Cat# CYT-MM-MRD8-R, (see manufacturer's instructions)
• multiepitope CD38-FITC
• Anti-CD56-PE (clone C5.9)
• Anti-CD45-PerCP-Cyanine5.5 (clone EO1)
• Anti-CD19-PE-Cyanine7 (clone 19-1)
• Anti-CD117-APC (clone 104D2)
• Anti-CD81-APC-C750 (clone M38)
• multiepitope CD38-FITC
• Anti-CD56-PE (clone C5.9)
• Anti-CD45-PerCP-Cyanine5.5 (clone EO1)
• Anti-CD19-PE-Cyanine7 (clone 19-1)
• Anti-cytoplasmic polyclonal immunoglobulin (Ig) κ-APC goat
• Anti-cytoplasmic polyclonal Igλ-APC-C750

• Anti-CD27 Brilliant Violet 510, BioLegend Cat# 302836, RRID: AB_2562086, O323
• Anti- CD138 Brilliant Violet 421, BD Biosciences Cat# 562935, RRID: AB_2737904, MI15

Flow cytometry of cryopreserved BM samples
Anti-CD8-APC, BD Biosciences Cat#555369, RRID: AB_398595, RPA-T8, 1:30
Anti-CD3-APC-R700, BD Biosciences Cat#565119, RRID: AB_2744385, UCHT1, 1:50
Anti-CD45-APC-H7, BD Biosciences Cat# 641417, RRID: AB_2800453, 2D1, 1:20
Anti-CD4-FITC, BioLegend Cat# 344604, RRID: AB_1937227, SK3, 1:20
Anti-CD194-BV421, BD Biosciences Cat# 562579, RRID: AB_2737663, 1G1, 1:20
Anti-CXCR3-PECy7, BD Biosciences Cat# 560831, RRID: AB_2033944, 1C6, 1:20
Anti-CD196-BV605, BD Biosciences Cat# 562724, RRID: AB_2737747, 11A9, 1:20
Anti-CD152-PE, BD Biosciences Cat# 555853, RRID: AB_396176, BNI3, 1:20

Anti-CD8-BUV395, BD Biosciences Cat# 563795, RRID: AB_2722501, RPA-T8, 1:50
Anti-CD4-BUV737, BD Biosciences Cat# 612748, RRID: AB_2870079, SK3, 1:50
Anti-CXCR4-BV421, BD Biosciences Cat# 562448, RRID: AB_11153865, 12G5, 1:30
Anti-CD45RO-BV711, BD Biosciences Cat# 563722, RRID: AB_2744413, UCHL1, 1:50
Anti-CD69-FITC, BD Biosciences Cat# 555530, RRID: AB_395915, FN50, 1:10
Anti-CXCR3-PECy7, BD Biosciences Cat# 560831, RRID: AB_2033944, 1C6, 1:20
Anti-CCR7-APC, BD Biosciences Cat# 566762, RRID: AB_2869854, 2-L1-A, 1:50
Anti-CD3-APCCy7, BD Biosciences Cat# 557832, RRID: AB_396890, SK7, 1:100
Fixable viability dye efluor506 Thermo Fisher Scientific Cat# 65-0866-14 ,1:1000

FACS for bulkRNAseq
Anti-CD3-APCCy7, BD Biosciences Cat# 557832, RRID: AB_396890, SK7, 1:100
Anti-CD8-BUV395, BD Biosciences Cat# 563795, RRID: AB_2722501, RPA-T8, 1:50
Anti-CD4-BUV737, BD Biosciences Cat# 612748, RRID: AB_2870079, SK3, 1:50
Anti-CXCR3-PECy7, BD Biosciences Cat# 560831, RRID: AB_2033944, 1C6, 1:20
Fixable viability dye efluor506 Thermo Fisher Scientific Cat# 65-0866-14 ,1:1000

T cell in vitro cytokine assay
Anti-CD3-APCCy7, BD Biosciences Cat# 557832, RRID: AB_396890, SK7, 1:100
Anti-CCR7-APC, BD Biosciences Cat# 566762, RRID: AB_2869854, 2-L1-A, 1:50
Anti-CD45RO-BV711, BD Biosciences Cat# 563722, RRID: AB_2744413, UCHL1, 1:50
Anti-CD8-BUV395, BD Biosciences Cat# 563795, RRID: AB_2722501, RPA-T8, 1:50
Anti-CD4-BUV737, BD Biosciences Cat# 612748, RRID: AB_2870079, SK3, 1:50

TNFa-PECy7, BioLegend Cat# 502930, RRID: AB_2204079, MAb11, 1:100
IFNg-PE, BioLegend Cat# 502509, RRID: AB_315234, 4S.B3, 1:50
Il-2-BV421, BioLegend Cat# 500328, RRID: AB_10962947, MQ1-17H12, 1:50
Fixable viability dye efluor506 Thermo Fisher Scientific Cat# 65-0866-14 ,1:1000

Multiplex immunofluorescence
CD3 (Labvision. Germany. clone SP7), CD8 (Abcam, Cambridge, UK, clone SP16), MUM1 (Dako, USA, clone MUM1p), LAT1 (Abcam, Cambridge, UK, clone EPR17573) and CXCR3 (Abcam, Cambridge, UK, clone ab133420)

Validation

All antibodies used are commercially available, broadly established and validated by the respecitve manufacturers as indicated on their websites (see RRIDS above for respective websites for each antibody). In addition, used antibodies are used routinely in our laborarotory with reproducible results.

Plants

Seed stocks

NA

Novel plant genotypes

NA

Authentication

NA

Flow Cytometry

Plots

Confirm that:

☒ The axis labels state the marker and fluorochrome used (e.g. CD4-FITC).

☒ The axis scales are clearly visible. Include numbers along axes only for bottom left plot of group (a 'group' is an analysis of identical markers).

☒ All plots are contour plots with outliers or pseudocolor plots.

☒ A numerical value for number of cells or percentage (with statistics) is provided.

Methodology

Sample preparation

After obtaining informed written consent, bone marrow samples were collected from iliac crest aspirations. Mononuclear cells from bone marrow and blood were isolated by density gradient centrifugation, frozen and stored in liquid nitrogen until further use.
For sample preparation, samples were thawed in a water bath at 37°C and transferred dropwise into RPMl-1640
10% FCS. Cells were centrifuged for 5 min at 350g and washed once with RPMl-1640 10% FCS. Cells were then resuspended in FACS buffer (FB, PBS 5% FCS 0.5 mM EDTA) containing fluorochrome conjugated antibodies, dead cell exclusion dye and Fc-receptor blocking solution. Cell suspensions were incubated for 15 min at 4°C in the dark. Cell suspensions were then washed with FB and resuspended in 0,2- 1 ml FB and were acquired using the respective analyzer or cell sorter.
Flow cytometry for detection of minimal residual disease (MRD) in fresh human BM samples was performed according to the highly standardized flow cytometry approach developed and described by the Spanish Myeloma Collaborative Group using a commercially available EuroFlow 8-color 2-tube MM MRD Kit (Cytognos, Salamanca, Spain).

Instrument

BD FACSFortessa flow cytometer
BD FACSLyric flow cytometer
FACSAria Fusion or FACSAria II equipped with 100µm nozzles

Software

FACSDiva & FlowJo v10.7.1 (BD Biosciences, San Jose, CA, USA)
Infinicyt 2.0 (Cytognos, Salamanca, Spain)

Cell population abundance

For single-cell RNAseq, sorted cell numbers were confirmed using a LUNA automated cell counter (Logos Biosystems). A volume of 33.8µl of the cell suspension was used as input without further dilution or processing, with final concentrations around 300cellsper µl.

Gating strategy

FSC-SSC gates were set so that FSC low and SSC high cells were excluded, following by singlet gating using FSC-A vs. FSC-H. After doublet exlcusion, dead cells, which are efluor506 high were excluded according to the manufacturer's instructions and the indicated gating strategies (see respective figures that show flow cytometry data and corresponding method sections) were followed. During sorts for single cell RNA sequencing, vibrant dye was used for dead cell exclusion as shown in Supplemental Figure 1.

☒ Tick this box to confirm that a figure exemplifying the gating strategy is provided in the Supplementary Information.
